# Evaluation of a Novel Cardiology Undergraduate Medical Education Curriculum

**DOI:** 10.7759/cureus.27360

**Published:** 2022-07-27

**Authors:** Garred S Greenberg, Mayce Mansour

**Affiliations:** 1 Department of Medicine, Icahn School of Medicine at Mount Sinai, New York, USA

**Keywords:** medical education, cardiology, internal medicine training, internal medicine-cardiology, flipped-classroom, undergraduate medical education, medical education curriculum

## Abstract

Introduction

Cardiology is a complex discipline that requires mastery of key principles and the ability to apply them in varied clinical scenarios, which may be challenging to teach in the traditional lecture-based format. The purpose of this educational intervention was to evaluate the effect of a flipped classroom model on knowledge base and attitudes towards high-yield cardiology concepts in third and fourth-year medical students at our institution.

Methods

An invitation to this optional course was sent to third and fourth-year medical students at the Icahn School of Medicine at Mount Sinai. Interested students were sent a document providing optional pre-course self-directed educational materials designed to take one hour to review. The materials included videos, graphics, and short sections of articles related to heart failure (HF), acute coronary syndrome (ACS), and tachyarrhythmias (TA). Students were then scheduled for a thirty-minute small-group session with a clinician, during which they reviewed the diagnosis and management of HF, ACS, and TA on an online video conference platform. Anonymous pre- and post-course assessments to measure knowledge and confidence were collected.

Results

Twenty-one students completed the pre-course assessment, and 19 students completed the post-course assessment. Seventy-nine percent of the students reported completion of at least half of the self-directed pre-work. The average score on the knowledge assessment rose from 42% to 71% after the course (p<0.001). After the course, 18 (95%) felt comfortable contributing to the management of a case of HF, 16 (84%) a case of ACS, and 13 (68%) a case of TA.

Conclusion

Knowledge assessment scores and learner self-confidence with the management of HF, ACS, and TA rose significantly after undergraduate medical education students completed this flipped classroom training. This exploratory study showed that the flipped classroom model with small group sessions can be a well-received model for medical student cardiology education among a self-motivated group of learners, though further analysis with a larger learner cohort is needed.

## Introduction

Engaging educational models, such as interactive learning modules and flipped classroom approaches, are becoming more popular in undergraduate medical education (UME) [1-4]. The flipped classroom model (FCM), which involves having students learn topics independently and asynchronously and then utilizing time with their instructor to reinforce knowledge and practice critical thinking, has been put forward as a new approach that warrants investigation for medical school education [5]. While studies of the FCM in medical education are difficult to compare given heterogeneity, a meta-analysis of FCM use in health professions education shows that, overall, this approach is associated with improved outcomes on assessments and greater satisfaction as compared to more traditional classroom models [6].

The potential benefits of the FCM include flexibility, increased educator efficiency, and increased learner engagement [7-8]. Given that medical students have significant time constraints during training, flexible home self-learning is appealing. The class time is designed to utilize a higher level of Bloom's taxonomy cognitive processes, such as evaluating and analyzing clinical cases [9].

Cardiology is a complex field that requires an in-depth level of knowledge about various medical conditions and the ability to apply that knowledge to complex and nuanced clinical cases. The FCM lends itself to the teaching of cardiology because it allows learners to learn the fundamentals on their own time while reserving time spent with a clinician to work together to solve clinical cases. The FCM has been highlighted as an important new model to improve lifelong learning in the field of cardiology [10-11]. One institution used the FCM to teach cardiology topics to 98 internal medicine residents by assigning readings from a cardiology textbook to be completed at home, followed by an instructor-led review of clinical cases in small groups [12]. While this study did not find any difference in knowledge between the FCM and the control group, it was likely because adherence to the weekly readings (51%) and faculty sessions (35%) was quite low. For graduate medical education, it has been noted that the increased time constraints from clinical duties would be a difficult challenge to the implementation of the FCM [13]. There is limited evidence of FCM utility at the UME level, as medical students typically have more flexible time for self-directed learning than residents.

The goal of the present study is to assess the impact of a custom-made, single-session FCM on the learning of three high-yield cardiology topics in the UME setting at our institution, as assessed by a self-assessment and a knowledge assessment.

## Materials and methods

Study design

A pre-post study design was used to assess the impact of the educational intervention, with electronic assessments conducted before and after the intervention. The intervention was designed to require ninety minutes to complete, utilizing an asynchronous self-learning component focused on knowledge and comprehension of the selected cardiology topics, followed by a synchronous small group session focused on the application of the knowledge to clinical cases.

Learners completed a pre-course assessment before starting self-directed learning materials, as well as a post-course assessment within a week of finishing their assigned small group session. The two assessments were optional, anonymous, and did not collect any identifying information. The assessments measured satisfaction with the Icahn School of Medicine at Mount Sinai (ISMMS) cardiology curriculum, satisfaction with this pilot cardiology curriculum, learner confidence in select cardiology topics, and learner knowledge. Knowledge was assessed with a brief quiz on clinical scenarios, and the same quiz was used for the pre and post-course assessments.

Volunteers were asked to complete self-directed learning materials, including multimedia, articles, and graphics focused on three core cardiology topics: heart failure (HF), acute coronary syndrome (ACS), and tachyarrhythmias (TA) (see Appendix). The self-directed materials were designed to take one hour to complete. They were marketed as being optional given the time constraints of medical school education.

The students were scheduled for small group sessions, which were conducted via online video-conferencing. The sessions were all led by the same educator, were timed to last approximately thirty minutes, and had an average of three students per session. The small group sessions were focused on applying the core knowledge gained from the self-directed materials to clinical cases. The educator asked direct questions in a sequential manner to guide learners through the clinical manifestations, diagnosis, and management of key cardiology topics including HF, ACS, and TA.

Participants and setting

An email invitation to this optional medical education course was sent out to the entire class of third-year and fourth-year medical students at the ISMMS. Inclusion criteria required participants to be over 18 years old, and be medical students within the ISMMS. The sample is a convenience sample of twenty-one students who volunteered to participate. Informed consent documentation was provided (see Appendix).

Analysis

Descriptive statistics were compiled from assessment results. An unpaired t-test was used to compare pre-course and post-course performance on the knowledge assessment. The Mann-Whitney U test was used to compare pre-course and post-course self-assessment of skill. Statistics were performed using R [14]. This study was reviewed by the Mount Sinai Institutional Review Board and was considered exempt research. Participation in both the surveys and educational sessions was anonymous and optional.

## Results

Twenty-one medical students scheduled completed the pre-course assessment, and 19 students completed the post-course assessment. Of the students who completed the pre-course assessment, 13 (62%) were third-year students, and eight (38%) were fourth-year students. Of the students who completed the pre-course assessment, 10 (48%) were satisfied with their current cardiology curriculum.

After completing the curriculum, 18 students (95%) were satisfied or very satisfied overall with the pilot FCM cardiology course (Table 1). Satisfaction was high with both the self-directed component (17 students, 89%) and the small group case component (18 students, 95%). All students felt that the course was clinically applicable, and all students felt that having the small group session on their schedule motivated them to complete the self-directed materials beforehand. Fifteen (79%) of the students completed at least half of the self-directed materials.

**Table 1:** Student satisfaction with flipped classroom model course Student degree of satisfaction with the self-directed component, the small-group component, and the overall course (n=19).

	Self-directed	Small-group	Overall
Very dissatisfied	0%	0%	0%
Dissatisfied	0%	0%	0%
Neutral	11%	5%	5%
Satisfied	32%	32%	32%
Very satisfied	58%	63%	63%

The average performance on the knowledge assessment rose from 42% to 71% after completion of the course (p<0.001). Before the course, only 19% felt comfortable contributing to the management of a case of HF, 19% felt comfortable contributing to the management of a case of ACS, and none felt comfortable contributing to the management of a case of TA. After the course, this self-assessed comfort rose to 95% for HF, 84% for ACS, and 68% for TA (Table 2).

**Table 2:** Self-assessed student confidence Mean confidence score, rated on a five-point Likert scale (1 - very unconfident, 5 - very confident), both prior to the course (n=21) and after the course (n=19). HF - heart failure; ACS - acute coronary syndrome; TA - tachyarrhythmias

	Pre-course	Post-course	p-value
HF	2.57	4.21	p<0.001
ACS	2.62	4.00	p<0.001
TA	1.95	3.79	p<0.001

## Discussion

The FCM is a strategy that has been shown to encourage the use of higher levels of thought process and provide enhanced student engagement in multiple educational settings. By allowing students to cover basic material on their own time and at their own pace, time with instructors can be reallocated to activities that involve the application of knowledge, critical thinking, and problem-solving. This model lends itself to the effective teaching of clinical cardiology, given that mastery of the topic requires both a strong base of knowledge and experience with applying the knowledge to real-world cases.

Various educational models are employed at the UME level; however, historically, the bulk of UME education comes in the form of large group lectures, where core information on medical topics is presented in a fashion that may limit engagement. According to the Association of American Medical Colleges (AAMC) 2021 survey of second-year medical students, only 28.6% of respondents attend in-person pre-clerkship lectures most of the time, and only 43.1% of respondents attend virtual pre-clerkship lectures most of the time [15]. The implementation of novel educational models is an important tactic by which educators can increase medical student engagement and improve the quality of their learning. While the flipped classroom model has been tested in other populations and other topics, our literature review did not reveal a study of its use for teaching cardiology to UME learners, so our study addresses a need.

In our study, we found excellent improvement in a knowledge assessment among medical students participating in FCM educational intervention regarding core cardiology topics. Additionally, satisfaction was quite high amongst the students in the intervention. After the course, which is designed to be brief in order to improve engagement, students felt more confident in basic cardiology topics and felt what they had learned was clinically applicable.

There are multiple notable limitations to this study. Because this was an optional course, the students were self-motivated, and the study is subject to selection bias. Learner demographics were not assessed to preserve anonymity. The same educator proctored all the small-group sessions, and students all joined from a single institution, limiting generalizability. The knowledge assessment was brief, and the educator was not blinded to the questions. Additionally, the knowledge assessment was administered immediately after the intervention, so retention of the material presented is unclear. Further evaluation of this course could involve multiple educators, a broader subset of learners, or another knowledge assessment several months after the intervention. Future studies could aim to assess the broader use of the FCM in other topic areas as an adjunct to or replacement for traditional lecture-based formats.

## Conclusions

Our study demonstrates that utilizing the flipped classroom model with small group sessions for cardiology education at the undergraduate medical education level can be well-received and effective at improving learner knowledge and self-confidence among a self-motivated population of learners. With an intervention requiring ninety minutes of time, and only thirty minutes of active educational time, students performed significantly better on a knowledge assessment, felt more comfortable with basic cardiology topics, and reported high degrees of satisfaction. This was an exploratory study, and further analysis with a larger learner cohort is needed.

## Appendices

This course is a pilot for how to standardize the teaching of high-yield cardiology topics for medical students.

With this optional course, I hope to help you learn the basics of management of three important cardiology topics - acute coronary syndrome, heart failure, and tachyarrhythmia. The approach will be multifaceted and will start with self-teaching with a few short online videos and parts of articles. Then, students will meet in small groups for a session with an educator to go through practice cases. This will hold students accountable for preparing adequately and give them a chance to apply their knowledge.

The whole course is designed to take less than two hours in total (~one-hour flexible self-study, a 30min session, and a pre/post assessment). All parts of the course are optional.

Curriculum

Take the pre-course survey and quiz (<5 minutes).

Learn about acute coronary syndrome (less than 25 mins total). Watch this short online MedEd video (can watch on 1.5x speed) https://www.youtube.com/watch?v=pzM-uOOYccQ. See the Acute MI review from NEJM (attached) - this focuses on the management of Type 1 MI. If you're tired, focus on the graphics/tables. See Figure 1 to notice the difference in pathophysiology between Type 1 and Type 2 MI. Read the "Univ Definition of MI" from Circulation (attached). Specifically, I would focus on Section 2, Section 6, and Sections 7.1 to 7.3. (three pages in total). Try to notice the difference between Type 1 or 2 MI and "myocardial injury."

Learn about heart failure (less than 20 mins total). Watch this great video from the Cleveland Clinic (can watch on 1.5x speed). Make sure you understand how "wet" and "cold" changes management https://www.youtube.com/watch?v=Z3akxkoetRo. Check out some of the figures in the 2019 ACC article on patients hospitalized with heart failure (attached). It's too long to read - I would focus on Tables 2 and 3, Figure 3, and Figure 6 (to understand diuretic dosing).

Learn about tachyarrhythmias (less than 20 mins total). Look at the UpToDate algorithm (attached) to learn the differential of wide vs. narrow and regular vs. irregular tachyarrhythmias. Think about the difference in management between a stable and an unstable or pulseless patient. Watch this awesome video by Strong Medicine on 1.5x ttps://www.youtube.com/watch?v=pd5FF8jGR78. Consider this video if you want more practice https://www.youtube.com/watch?v=NULY6Ecj6gA

We will schedule a 30min fast-paced session to go through cases that will allow you to apply your knowledge. Post-course survey and quiz, <5 mins, link to be distributed after our group session.

-----------------------------------

The purpose of this research study is to see if this style of teaching cardiology topics is engaging, satisfactory, and effective.  You are being asked to take part in a research study because you are a medical trainee who volunteered to participate.

Being in a research study is completely voluntary. You can choose not to be in this research study. You can also say yes now, and change your mind later. Your decision on whether or not to participate will not affect your academic or professional standing.

If you agree to take part in this research, you will be asked to optionally complete self-directed learning materials and join in an optional ~ 30-minute small group learning session. Your participation in this study will take less than two hours in total. The optional self-directed learning takes ~ one hour, the optional small group session takes ~30 minutes, and the pre and post-survey will take ~ five minutes each. We expect that 100 people will take part in this research study.

You can choose to stop taking the survey at any time. You must be at least 18 years old to participate. If you are younger than 18 years old, please stop now.

The possible risks to you in taking part in this research are:

§  Spending two hours in an ineffective educational program

The possible benefits to you for taking part in this research are:

§  Improving education at your institution

§  Learning about helpful cardiology topics

To protect your identity as a research subject, no identifiable information will be collected (we will not collect your name or any identifiers). In any publication about this research, your name or other private information will not be used.

If you have any questions about this research, please contact the Researcher at . You can also call the Program for the Protection of Human Subjects Office at 212-824-8200. This project was determined to be exempt from federal human subjects research regulations.
